# Characteristics and outcomes of referrals to CAMHS for children who are thinking about or attempted suicide: A retrospective cohort study in two Scottish CAMHS

**DOI:** 10.3389/fpsyt.2022.914479

**Published:** 2022-09-01

**Authors:** Lynne Gilmour, Catherine Best, Edward Duncan, Margaret Maxwell

**Affiliations:** NMAHP-RU, University of Stirling, Stirling, United Kingdom

**Keywords:** suicide, children and young people (CYP), adolescents, CAMHS, suicidality, mental health, pathways of care

## Abstract

Suicide among children and young people (CYP) is a leading cause of death. In the UK children identified as suicidal are referred to Child and Adolescent Mental Health Services (CAMHS) for assessment and treatment. However, the number of children referred for suicidality, and their care journey is unknown. This retrospective cohort study conducted in two distinct CAMHS teams, in Scotland, UK, aimed to quantify the numbers of children referred for suicidality, describing this population and the outcomes of these referrals. All CAMHS referrals (*n* = 1159) over a 6-month period (Jan-June 2019) were screened to identify those referred primarily for suicidality. Data extracted included: age, gender, source of referral, reason for referral including underlying issues, whether offered an assessment, and referral outcome. Area based deprivation scores were attached to each referral. Associations between the referred CYP's characteristics (including source of referral and underlying issues) and referral outcomes were explored using Chi Square, Fishers Exact test, and one-way ANOVA. Referrals for 284 children were identified as being for suicidality across the two sites (Site A *n* = 104; Site B *n* = 180). These represented 25% of all referrals to these CAMHS over a six-month period. One third of these concerned children under 12. The underlying issues, referrals sources, and demographic indicators were similar in both sites. In site A 31% were offered an assessment, whilst in Site B which had a dedicated team for suicidal CYP, 82% were offered an assessment. Similarly, more children in Site B were offered treatment (47.8%), than Site A (7.7%). Referrals from A&E were prioritized in both areas, and those who had attempted suicide offered an assessment more often. Older children were more likely to be offered treatment, although they were more likely to present with a history of self-harming behavior and/or previous suicide attempt. There are high numbers of children being referred to CAMHS for suicidality, and many are young children (<12). There is variation within and between services in terms of assessment, referral outcomes and care pathways for these children. Having a dedicated team to respond to referrals for suicidality appears to support access to assessment and treatment.

## Introduction

Suicide is a leading cause of death of children and young people globally (1). National statistics in the United Kingdom reveal the numbers of suicides amongst those under 25 has been continually rising since 2017, with a marked 22% rise in the year 2018 (2). Childline (a UK wide telephone counseling service for children) report that 67 children a day called their helpline in 2018/19 for help with suicidal feelings, and there was an 87% increase (from 2015/16) in calls from children under 11 seeking help with suicidal thoughts and behaviors (3). The problem is a global issue (1, 4), as well as within the UK. Notably, Scotland is reported to have the highest rate of suicides amongst children in the UK^1^ (6).

Alongside these worrying statistics and commentaries, we are told that child and adolescent mental health services (CAMHS) are “not fit for purpose” (7). There have been reports describing them as the “Cinderella service”, underfunded in relation to physical health services (8, 9).

Demand for CAMHS services continues to grow; the umbrella of what is considered the remit of child and adolescent mental health services steadily widening (10). As a result, CAMHS services are currently under immense pressure, with demand for their services exceeding capacity (11–14). Pre Covid, one in five referrals to CAMHS in Scotland were rejected (15), and in England one in four were rejected (16). Since the pandemic, numbers of referrals have continued to rise and waiting times for many children and young people are over of a year (12, 15, 17). The number of these referrals that were made for children who are suicidal is unknown.

Various reports and reviews have considered the problem of CAMHS demand (14, 15, 18–20), although delivery of services and investment varies across as well as within the different countries that comprise the United Kingdom.

CAMHS in Scotland continues to operate a tier system of care in which CAMHS remain positioned as a specialist service, funded by the NHS. However, there has also been an increase in mental health support services in schools, and a continued shift in focus toward prevention and early interventions (14, 21). Recent reports suggest a re-design of mental health services will follow, making them more accessible with community-based “one-stop” service provision (14, 21). This has yet to be realized and attempts to better manage referrals to CAMHS have had little impact to date.

There is a paucity of reliable data from CAMHS generally and in Scotland the lack of available information was identified as barrier to service re-design (22). Public Health Scotland (PHS) [previously the Information Services Division (ISD)] collects CAMHS data from each health board (national workforce and performance data) which is limited to referral numbers and waiting times. Recent routine reports on waiting times indicate health boards are working to improve the accuracy of the data they provide to ISD (11). Information is still not routinely collected on the reason for referral. Therefore, the number of children who have been referred to CAMHS for reasons of suicidality and subsequently placed on a waiting list is undetermined.

In 2017, as a direct recommendation made in the (23) “National Mental Health Strategy” ISD and SAMH (Scottish Association for Mental Health) were commissioned to conduct an audit of rejected referrals to CAMHS (15). ISD collected quantitative information from seven participating health boards (7/14) about CAMHS referrals they had received and processed over 1 month (February 2018), whilst SAMH conducted an on-line survey, focus groups and telephone interviews with young people, parents and carers, GPs, and teachers. They found that 20% of all referrals to CAMHS were rejected. As part of the audit data set ISD requested information from the participating health boards about the reason a young person had been referred. They found there to be inconsistencies between the information provided by the boards, compared with the reasons given by children and families as to why a referral had been made. The data from the health boards showed 0.4% of referrals to have been made because of suicidal ideation, and 1.4% following self-harm, while suicidal ideation was one of the most cited reasons for referrals being made by the patients and families, revealing potential discrepancy in the figures provided. The investigators also queried the reliability of the data they were provided (15). While this audit provides valuable insight into the referral process overall and the extent of the problem in relation to rejected referrals from CAMHS, it does not adequately address the issue of quantifying the numbers of children who are suicidal or provide insight into the pathways of care they experience thereafter.

Also following the recommendation made in the “National Mental Health Strategy,” (23), in response to increased number of referrals to CAMHS (22% from 2013/14 – 2017/18) and increased waiting times on access to CAMHS, the Scottish Government commissioned a national audit of CAMHS services (22). This audit focused on the funding and efficacy of CAMHS. It used mixed methods, including routinely collected data (from ISD) alongside interviews and focus groups with patients and their parents / carers, senior staff, front-line staff, NHS managers and government representatives. Child and adolescent mental health services were not found to be easily accessible to children and young people, with different services and protocols in place in different areas. The Accounts Commission found there were large inconsistencies and variations in the funding, organization, and delivery of CAMHS services across the country. They reported it was not possible to accurately quantify local health board spending on CAMHS services, and that existing data on CAMHS outcomes was deficient. They described CAMHS as being under increasing pressure, with higher numbers of referrals and increasing waiting times. Despite the audit activity taken place in recent years, much remains unknown about the numbers of children who are referred to CAMHS for suicidality and how they are being managed.

This paper reports the findings of a retrospective cohort study documenting the numbers of children referred to CAMHS for suicidality, and the outcomes of these referrals in two Scottish CAMHS teams. It aimed to:

1) Quantify the numbers of children referred to two different CAMHS services in Scotland over a 6-month period for reasons of suicidality and document the outcome of these referrals.
2) Provide descriptive demographic information about the identified sample population: age, gender, family composition, etc.
3) Explore whether there is any potential relationship between reason for referral, referral source and demographic indicators with referral outcomes.

## Materials and methods

### Context / setting

The two selected CAMHS teams are in geographically different areas (Health Board regions) of Scotland, and are referred to throughout as site A, and B. They were selected as they offered understanding of contrasting environments, remote rural, and a mixture of accessible-rural / urban areas. They also reflect different CAMHS structures: within the CAMHS team in site A there are psychological services, learning disability and autistic spectrum disorder services, a looked after and accommodated nurse service, a core CAMHS nursing service, a Tier 4 CAMHS outreach team, and consultant psychiatrists; in site B there are core CAMHS nursing teams, an intensive support team, a looked after and accommodated team, a specific service for children who have experienced child sexual abuse, a suicide and self-harm team and consultant psychiatrists. Psychological services and autistic spectrum disorder and learning disability teams sit out with CAMHS within Site B. The intense nature of on-site review of all referrals data and study resources precluded investigation of more sites.

### Data source

Information regarding the number of children who are referred to CAMHS primarily for reasons of suicidality is not routinely available. This information can only be identified from the initial referral letter and / or completed referral form sent to the CAMHS service. These referral forms and letters were identified as the data source for this study as they provided a means to identify the sample population. In site A all referrals were stored in paper-based files, while in site B, PDF copies of these referral letters and forms were held electronically.

Data were collected from referrals made over a 6-month period: January –June 2019. In Site A, a total of 397 referrals were screened. This was the total number of referrals received by CAMHS at Site A (January and June 2019). This comprised: referrals that were accepted and put on the waiting list (*n* = 161); rejected referrals (*n* = 209); and direct tier four referrals (usually accessed by presentation at A&E) (*n* = 27). These categories reflect how referrals were organized within CAMHS at Site A. The total number of referrals screened in Site B was 762. This was the total number of referrals made to CAMHS across Site B (Jan–June 2019), that were directed to the following services: Suicide and Self-harm team (*n* = 131), East (*n* = 226), West (*n* = 294), specific services for children who are looked after and accommodated, and for children who have experienced child sexual abuse (*n* = 111).

Referrals were eligible to be included in the study if they stated within them that the primary reason for the referral being made was that the child had been thinking about or had attempted suicide. The anonymised data set was then entered into an SPSS file, to enable predefined descriptive and inferential statistical analysis to be conducted.

### Variables

The data extracted reflected the study objectives. Variables were pre-specified (Supplementary Table 1: Retrospective cohort study variables). Reasons for referral, referral outcomes and anonymised demographic data were extracted to provide descriptive statistics for the sample population as well as explore the potential relationship between demographic indicators and referral outcomes. For example, the Scottish Index of Multiple Deprivation (SIMD) 2016 version was used to calculate a deprivation score for each child referred for reasons of suicidality. The SIMD tool ranks geographical data zones (based on postcodes) by their level of deprivation. Decile rankings are achieved by combining data from 7 domains of deprivation measured: income, employment, health, education, access, crime, and housing.

### Data extraction

The data were extracted in person by LG, from within the CAMHS sites, according to the study protocol. Extracting data to count frequencies from a qualitative source is not straightforward. Some degree of interpretation inevitably occurs. In this instance the presence of certain words / phrases in the referral document were read as indicative of suicidality. For example, “suicidal thoughts” or “been thinking about suicide” etc. However, the richness of the qualitative data is undeniably lost during this process, and coding uncertainty occurred as referral information was sometimes unclear or scant. Furthermore, variables such as referral outcome were not clearly defined within the individual records and narrative data was used to ascertain what happened to the referral (e.g., whether the individual was offered a face-to-face assessment, added to a waiting list etc.). It is important to note that categories were defined by the research team, based upon the information available, and were not necessarily categories employed by the CAMHS teams. Pooling data into categories meant that simple terms like “closed” were used to describe a collection of possible occurrences that could be counted as such. For example, “closed” was used to indicate the case was closed because the person did not attend appointment they were offered, attended one and did not engage thereafter, was not offered further treatment, or attended for treatment and this has ended. The back story as to why the case was closed was lost through the process of anonymizing and categorizing the data. Categories that define a range of situations are outlined in Supplementary Table 1: Retrospective cohort study variables. Missing data was recorded as such.

A coding diary was kept throughout the process, which allowed decisions to be tracked and to ensure consistency. Categories were discussed, agreed, and collapsed as necessary through discussion between LG, ED, and MM. Categories such as “other” were collapsed during the analysis process in consultation with CB, as numbers in these groups would have been so low, they may have compromised individual confidentiality.

### Analysis

Descriptive statistics were produced summarizing the characteristics of the children referred and their referral outcomes. Continuous variables were summarized as mean and standard deviation, or median and inter quartile range (IQR) as appropriate. Categorical variables were summarized as frequencies. Chi Squared analysis and Fishers exact tests were used to explore relationships between categorical variables. Fisher's exact test was employed when small cells sizes meant that Chi Square tests were not appropriate. One-way ANOVAs were used to examine if continuous variables such as age at referral were associated with referral outcome.

## Results

### Base line and demographic data

The total number of children identified as being referred to CAMHS because of concerns about suicidality in Site A was *n* = 104, which was 26% of all referrals reviewed (*n* = 397). Table 1 shows reason for referral was broken down as follows:

**Table 1:** Reason for referral (Site A & B).

	**Site A**		**Site B**	
	**Number**	**Percent**	**Number**	**Percent**
Ideation	40	38.5	73	40.6
Attempt	8	7.7	12	6.7
Ideation & Previous suicidal behavior and / or self-harm.	46	44.2	58	32.2
Attempt & Previous suicidal behavior and / or self-harm.	10	9.6	37	20.6
Total	104	100	180	100

The total number of children identified as having been referred to CAMHS because they were experiencing suicidality in Site B during this time was *n* = 180, which was 24% of all referrals screened^2^. A breakdown of the reason for referral as identified for those referred primarily for suicidality is provided in Table 1.

### Gender and age

**Figure 1:**
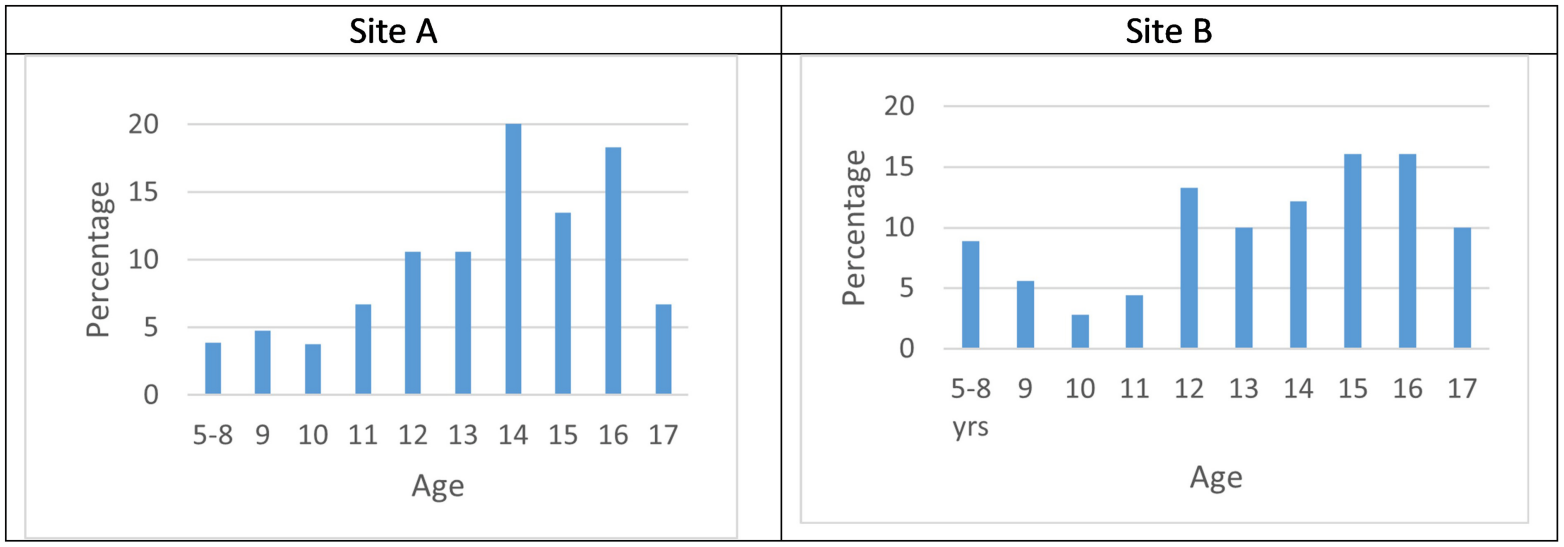
**(A,B)** Age at referral.

Of the children referred for suicidality to Site A; 42 (40.3%) were boys, 62 (59.7%) were girls. Their age at the point of referral ranged from 5–17. The mean age was 13.5, with a standard deviation of 2.52; Thirty percent of children were aged 12 and below. There was 1 missing data unit for age–therefore these statistics depicted in Figure 1A represent 103 referrals.

In Site B, 76 (42.2 %) of children identified were male, 104 (57.8%) were female. Age ranged from 5 to 17 years, with a mean age of 13.28, and a standard deviation of 2.96. Thirty-five percent of children were aged 12 and under. This is presented in Figure 1B.

### Family composition

**Figure 2:**
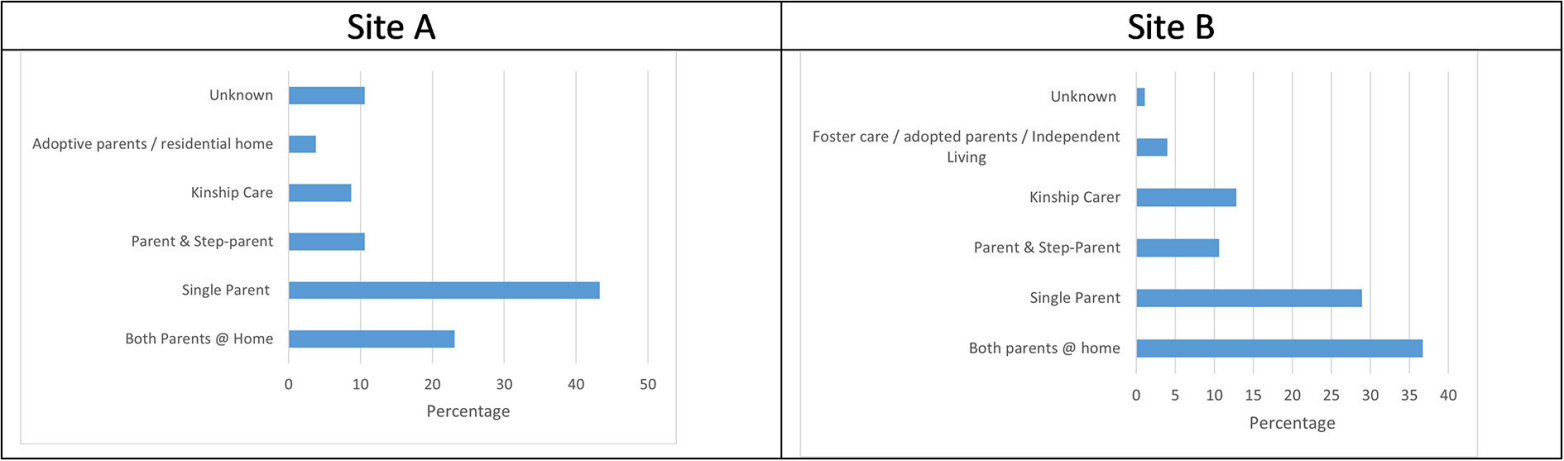
**(A,B)** Family composition.

As illustrated in Figure 2A most children referred for suicidality in Site A were found to live at home with at least one of their parents (77%), with the majority being single parent families. However, family composition was not described in 10% of the referrals. Similarly, Figure 2B shows most children in Site B lived with at least one of their parents (76.2%). However, in contrast to Site A, most children had both parents at home. Less than 5% of referrals lacked any information about family composition in Site B.

### SIMD (Scottish Index of Multiple Deprivation) data

**Figure 3:**
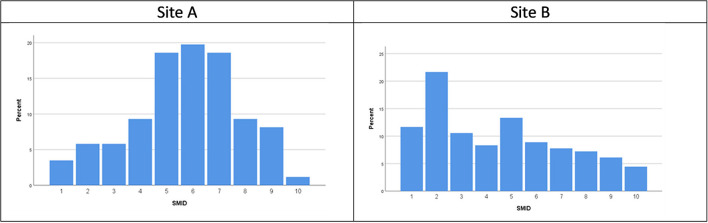
**(A,B)** Scottish Index of Multiple Deprivation (SIMD 16) data.

A deprivation score for each child identified as being referred for reasons for suicidality was calculated based on the Scottish Index of Multiple Deprivation (SIMD) 2016 (the 2020 coding was released following data collection). Postcode data was missing for 17.3% of referrals for suicidality in Site A. The spread of postcode decile for the remaining 86 individuals is illustrated in Figure 3A below. This is contextualized within the SIMD data for the region in the discussion which follows.

The SIMD data from the postcodes of the children referred to Site B illustrated in Figure 3B showed there was a high proportion living in the second most deprived areas (based on SIMD ranking) and fewer referrals were for children living in the more affluent areas.

### Occupation of referrer

**Figure 4:**
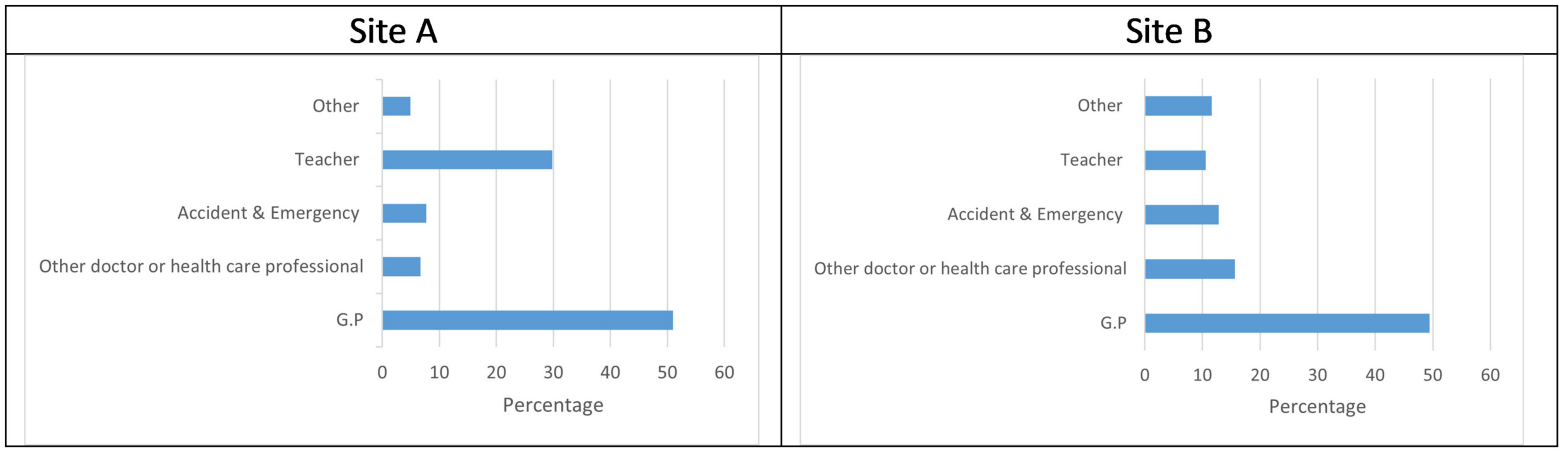
**(A,B)** Occupation of referrer.

Overall, as is shown in Figure 4A, 65% of referrals for children presenting with suicidality in site A were made by medical professionals, with 51% of these being GP referrals. The second largest source of referral were teachers (29.8%). Similarly, as can be seen in Figure 4B below, most referrals to Site B for children who were suicidal came from medical professionals (78%) but with higher numbers of referrals from “other doctor or healthcare professionals” and “A&E” than in Site A. Site B had fewer referrals from teachers (22%) than Site A, but a higher number of referrals from other (Other includes school nurse, social worker, other support organization and parents) sources (16%).

### Assessment

**Figure 5:**
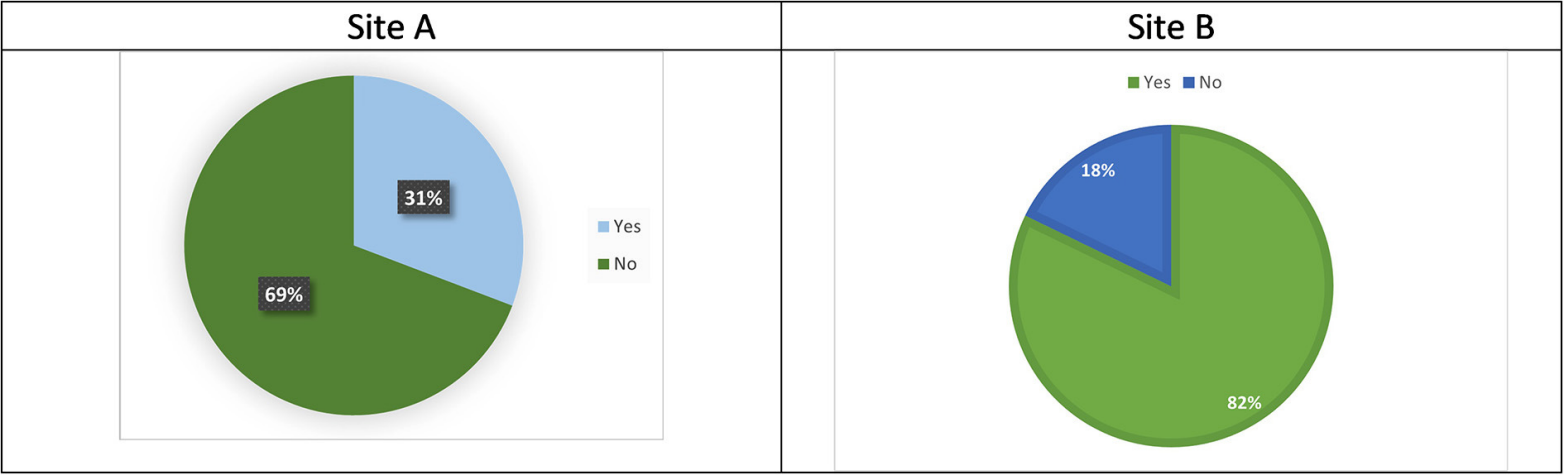
**(A,B)** Offered an assessment.

When a child is referred to CAMHs in Site A, they may or may not be offered a face-to-face assessment by a CAMHS worker, before their referral is rejected, redirected, or added to the waiting list etc. Assessment refers to a face-to-face appointment with a CAMHS clinician, it does not account for background work e.g., information gathering etc. to support the screening process. These assessment appointments generally involve some form or risk assessment, safety planning and exploration of family circumstances and any underlying issues. The format varies between clinicians and sites. Figures 5A,B shows that most (69%) children referred to CAMHS for suicidality in Site A were not offered a face-to-face assessment, though the practitioner screening the referrals may in some instances have provided a telephone consultation with the person making the referral. While in Site B, most children (82%) identified as having been referred for suicidality were offered a face-to-face assessment.

### Referral outcome

**Figure 6:**
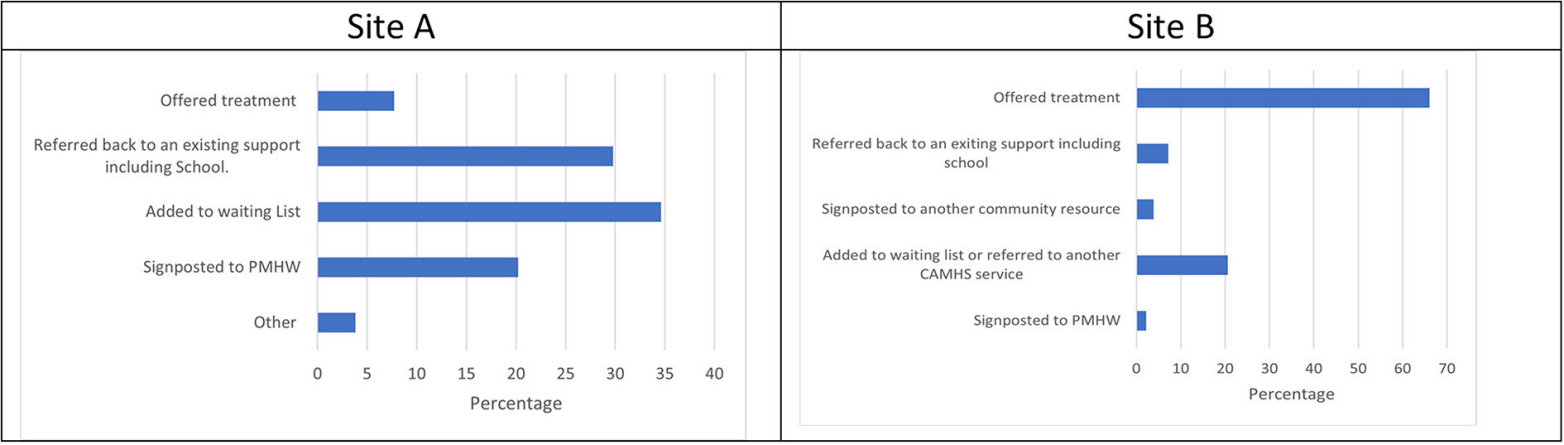
**(A,B)** Referral outcome.

The variable referral outcome documents the decision that was made immediately following referral and / or assessment in relation to whether the person was offered a service with CAMHS or not. Figure 6A shows that in Site A, <10% of children were offered treatment straight away, and although 34.6% were added to the waiting list, most referrals were not accepted (57.6%). In contrast, Figure 6B shows most children (66.1%) in Site B were offered treatment. One fifth (20.5%) of referrals were either added to a waiting list or referred to another CAMHS service such as primary care psychology, and 13.3% were signposted or re-directed.

### Underlying issues

**Figure 7:**
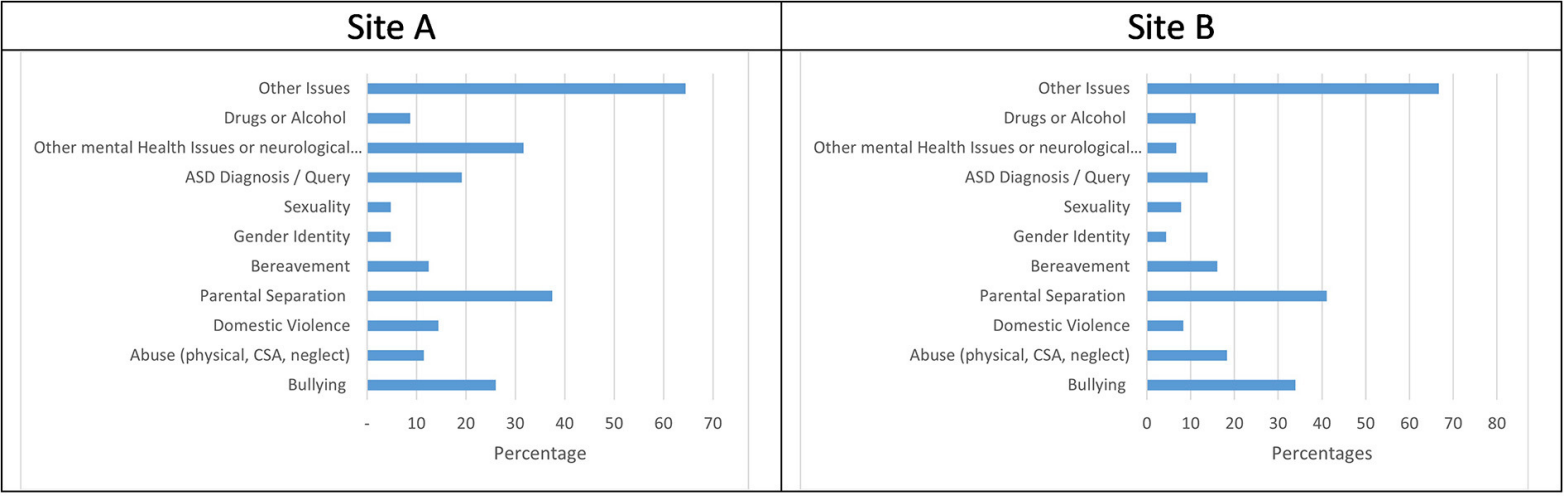
**(A,B)** Underlying issues.

Underlying issues were identified within the initial referral information or the first contact with the CAHMS service. Each child may have had more than one issue reported. Figure 7A shows that in Site A parental separation, other mental health issues or neurological condition, bullying, and ASD were the most common issues. In Site B the issues most reported for children were parental separation (41.1%) and bullying (33.9%), followed by abuse (18.3%) and bereavement (16.1%), shown in Figure 7B.

### Exploration of associations between demographic indicators and outcomes

#### Reason for referral and assessment

Fisher's exact tests were conducted to explore if there was any relationship between reason for referral and assessment. Although the numbers of children who had attempted suicide both with and without previous suicidal behavior was much lower than the number of children referred for suicidal ideation, they were more likely to be offered an assessment. A Fishers Exact Test (FET) confirmed there was a potential relationship between these variables in site A (*p* = 0.003 FET). Similarly, in Site B, a FET found there may be a relationship between reason for referral and assessment (*p* = 0.010 FET), as there were slightly higher numbers of children offered an assessment following a suicide attempt (with and without previous behavior). However, overall, most children in Site B were offered an assessment (see Figure 5).

#### Reason for referral and referral outcome

Unfortunately, it was not possible to reliably determine whether there was any association between the reason that a child had been referred, and the outcome of the referral for either site. This was because the large number of different reasons for referral meant that data was too sparse for statistical analysis. Categories had been collapsed as far as was conceptually feasible.

#### Referral source and assessment

A Fisher's Exact test (*p* < 0.001, FET) indicated there may be a relationship between the source of the referral and whether the child was offered an assessment. Over 85% of referrals from A&E were assessed although the overall number of those referrals was <10. While over 85% of G.P referrals were not assessed, albeit the greatest number of referrals received were from GPs.

Most children from whatever route of referral in Site B, were offered an assessment. However, there were some differences. For example, all children and young people in Site B referred by A&E were assessed, whereas <70% of referrals from teachers were assessed (*p* = 0.022 FET).

#### Referral source and referral outcome

Exploring the relationship between the referral source and referral outcome (n Site A with FET showed a potential relationship (*p* = 0.028). In Site B data was too sparse for such statistical analysis.

#### Age and referral outcome

A one-way ANOVA indicated a statistically significant difference between age and referral outcome (F _(4, 98)_ = 3.536, *P* = 0.010). The table of means [Table 2 (Site A)] shows that younger children were more likely to be referred to an existing support or onto a primary mental health worker.

**Table 2:** Age & referral outcome (Site A & B).

	**Site A**			**Site B**		
**Outcome of referral**	**Mean age**	**N**	**Std. deviation**	**Mean age**	**N**	**Std. deviation**
Signposted to PMHW	12.70	20	2.716	11.50	4	4.796
Added to waiting List	13.92	36	1.962	11.78	37	3.128
Other	14.86	7	1.676	11.86	7	4.100
Referred to an existing support including school	12.75	32	2.896	11.69	13	3.376
Offered treatment	15.50	8	1.512	14.06	119	2.423
Total	13.50	103	2.524	13.28	180	2.958

A one-way ANOVA also showed that there was a statistically significant difference between the age of children and their referral outcomes (F _(12, 167)_ = 2.964, *p* < 0.001) in Site B. From the table of means [Table 2 (Site B)] it appears children offered treatment are older than for other referral outcomes.

#### Age and reason for referral

Comparison of mean ages within reason for referral for Site A showed that the average age of children having attempted suicide and having attempted suicide with a history of suicidal behavior was slightly higher than for the ideation categories. A one-way ANOVA showed a statistically significant relationship between reason for referral and age (F _(3, 99)_ = 4.283, *p* = 0.007). In Site B one-way ANOVA, also showed that the relationship between the age of a child for each reason for referral category was statistically significant (F _(3, 176)_ = 2.990, *p* = 0.032).

### Summary of main findings

Approximately 25% of all referrals to CAMHS in both regions were for children presenting with suicidality (26% (*n* = 104) in Site A and 24% (*n* = 180) in Site B). The assessment and outcome of these referrals varied between the health boards. In Site A, 31% of children referred were offered an assessment appointment, compared with 82% of children in Site B.

Referral outcomes in Site A indicated that 8% of those assessed were offered treatment, 35% were added to the waiting list, 20% were signposted to primary mental health workers, and 37% of children were referred to other agencies, school or back to the referring agency for support. In Site B 48% of children referred for suicidality were provided and engaged in treatment, 18% of young people were offered treatment but did not engage or attend their appointment, 13% were added to the waiting list, 2% were signposted to primary mental health workers, 7% referred on to a different CAMHS service (e.g., psychology), 11% were referred to other agencies, school or back to the referring agency for support.

**Figure 8:**
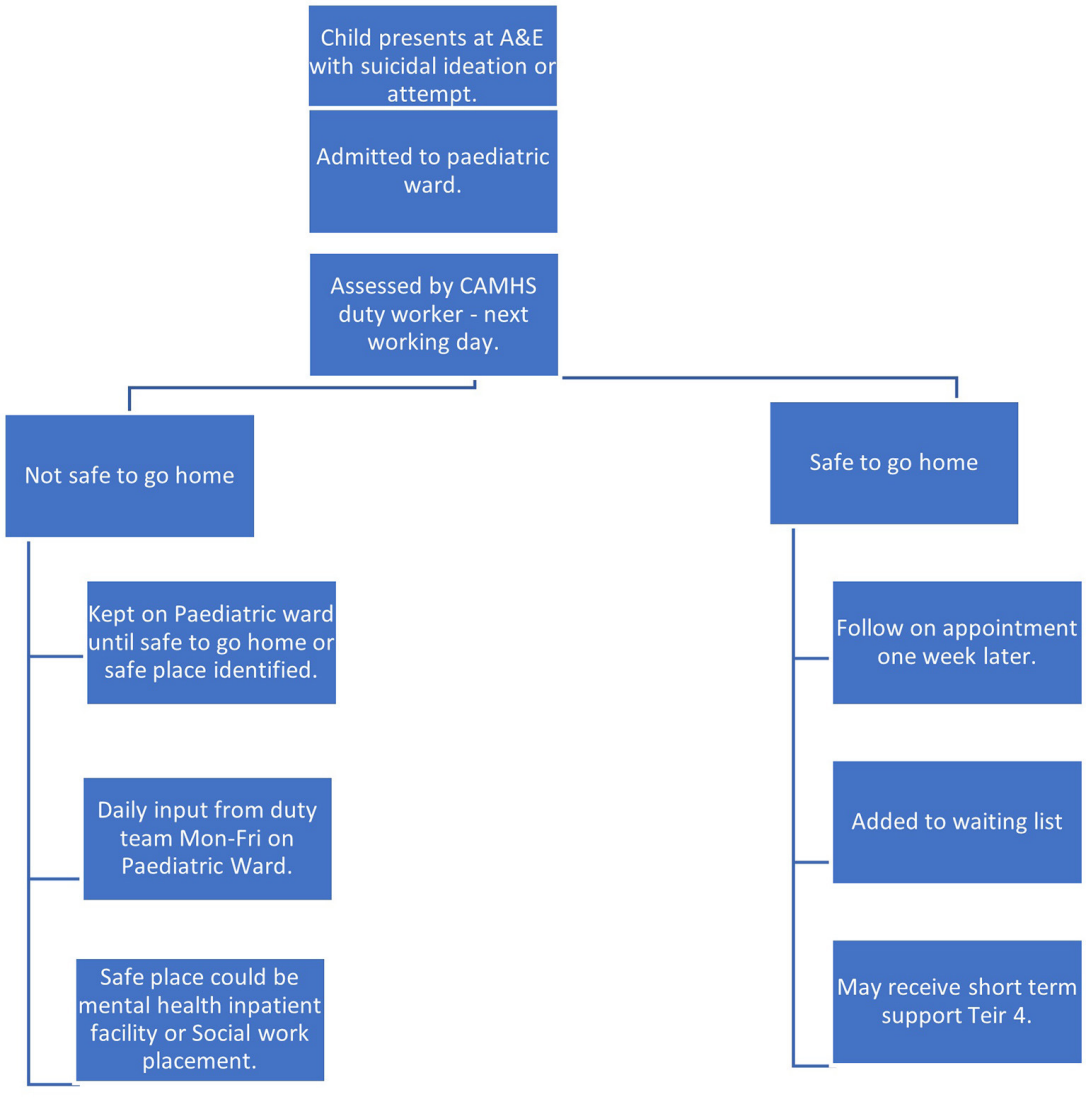
Referral pathway for children referred to CAMHS from A&E (Site A).

**Figure 9:**
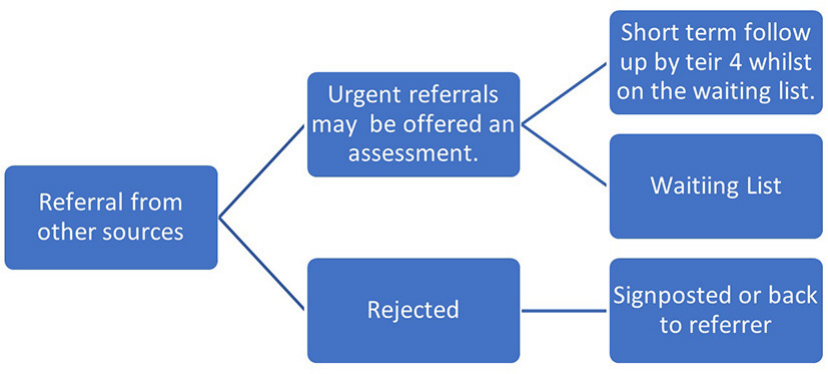
Referral pathway for children referred from other sources (Site A).

**Figure 10:**
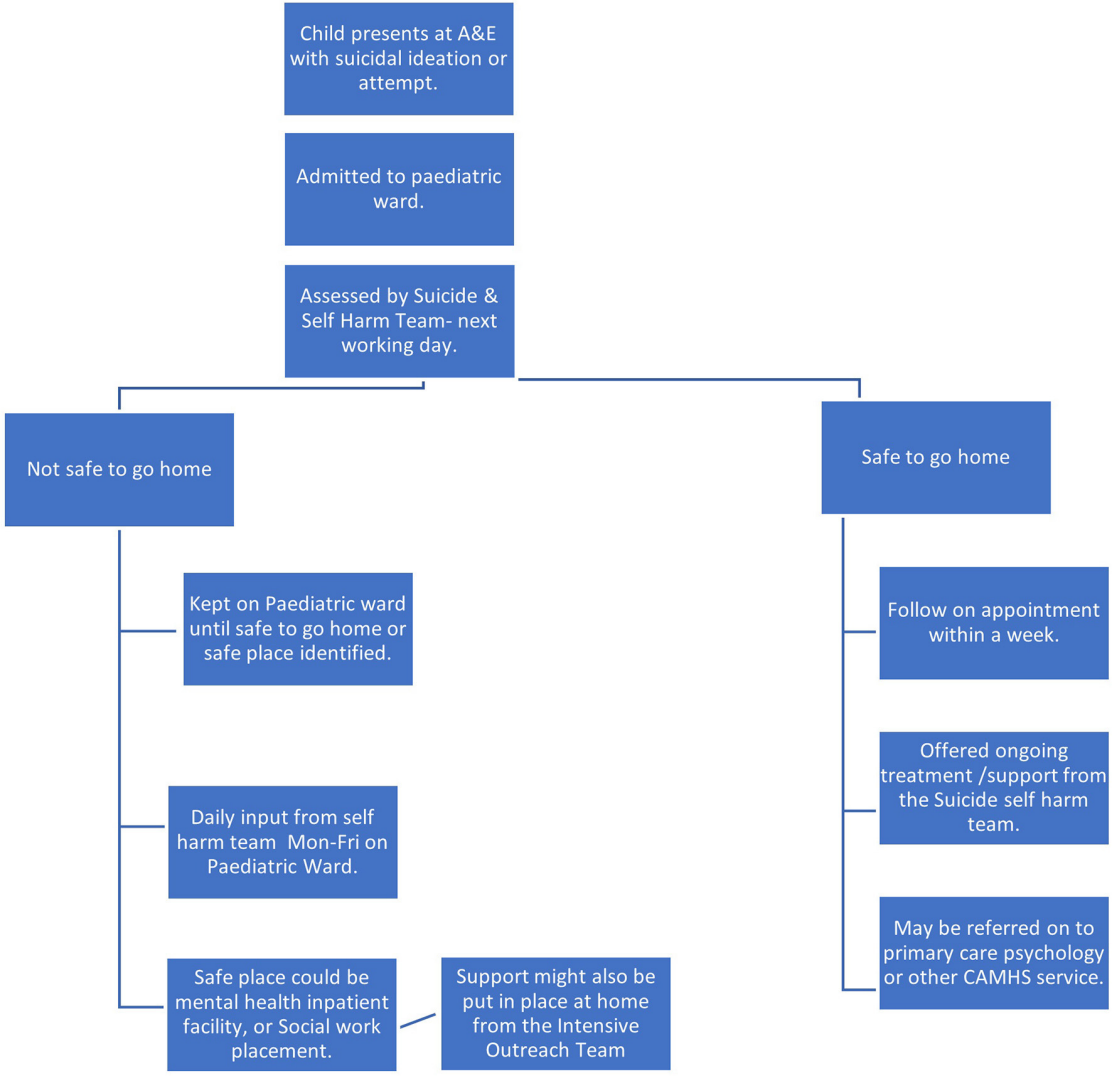
Referral pathway for children referred to CAMHS from A&E (Site B).

**Figure 11:**
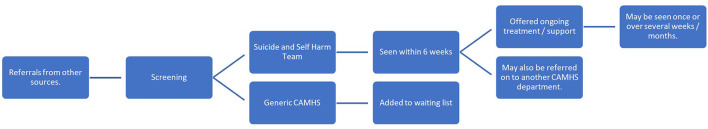
Referral pathway for children referred from other sources (Site B).

Through the process of data collection, it was possible to map the journeys of care made by children in the two sites following their referral to CAMHS. This is depicted in Figures 8–11, which follows.

The reason for referral, that is whether they had been thinking about suicide, attempted suicide, or had a history of suicidal behavior, may have had a bearing on whether children were offered an assessment in Site A and Site B. Children who had been referred following a suicide attempt being were offered an assessment more often than those referred for ideation. In both areas, children were also more likely to be offered an assessment if they had been referred directly from A&E than from any other referral source, although in Site B most children were offered an assessment regardless of referral source or reason for referral.

In both health board areas, there appeared to be a relationship between the age of the child and the reason for referral, although the nature of this relationship differed. In Site A the age of the children that attempted suicide seemed to be higher than those presenting with suicidal ideation. While in Site B the data suggested that older children were more likely to have a history of previous suicidal behavior than those being referred following a first attempt or suicidal ideation. In both services, the age of referral also seemed to have a similar relationship with the referral outcome–older children were more likely to be offered treatment. Demographic information from both areas indicated that the underlying issues identified in referrals, family composition and age range of these sample populations were generally similar.

Overall, this study shows that one quarter of all referrals to CAMHS in both sites were for children who had either attempted or been thinking about suicide, and that one third of these were for children under 12. The findings indicate that older children in both areas were more likely to be offered treatment. The underlying issues identified by referrers were similar in both health boards and included a broad range of complex familial and social factors, suggesting that suicidal children are not a homogenous group.

There was a considerable difference in the numbers of children who were offered assessments and treatment between the Sites reflecting the structural differences between teams. In Site A only 31% were offered an assessment, while in Site B, who had a specific suicide and self-harm team, were able to provide face to face assessments for 82% of children referred. Similarly, more children in Site B were offered treatment (48%), than in Site A (8%).

Our analysis suggests that having a specialist team to respond to referrals for suicidality may better equip CAMHS to assess children who have been referred due to suicidality and offer some form of intervention.

## Discussion

The two sites are situated in different parts of Scotland. Not only are the CAMH services operationally distinct from one another, but so too are the local cultural contexts. For example, the population of the geographical region of Site A in June 2019, was 235, 540 whilst in Site B it was 371,910. The number of children aged 0–15 years, in Site A and B was 39,335, and 64,473 respectively. While these numbers reflected a similar percentage of the locality's overall populations (16.7 and 17.3%), Site A has 39% less children aged 0-15 than Site B (24). This difference in child populations is reflected in the number of children referred to the CAMHS services, with Site A receiving 42% less referrals for children and young people who were suicidal during the data collection period.

The size of the population aged 16-18 is unknown as National Records Scotland data are grouped by ages 0-15 and 16–24 yrs (24). However, it is worth noting that although Site A only accept referrals for young people aged 16–18 yrs if they are still attending school, in Site B they work with all children up to 18 yrs. The percentage of referrals each CAMHS received for children aged 16-18 who were suicidal was 25 and 26% respectively. This shows that the difference in CAMHS remit for Site A had little impact on where services referred young people of this age. Or it could be there was a higher proportion of young people aged 16–18 years who were presenting to health services as suicidal in Site A (as we would expect there would also be a number referred to adult services), or there are higher numbers of children remained in school beyond 16 yrs.

The stark difference in the numbers of children offered face to face assessments between these regions, highlights the benefits in Site B having a discrete suicide and self-harm team. However, due to pressure on resources this team have since moved to a new model of care, which could adversely affect their ability to respond as quickly to these children in future.

The differences in numbers of assessments offered also reflects the geographical challenges faced by CAMHS workers in providing accessible face to face appointments for children living in remote and rural locations in Site A. Although there have been and are ongoing attempts to address this, through the provision of video linked “near me” appointments, and provision of primary mental health workers located within specific regions, these have presented challenges in themselves, and arguably it remains a gap in service provision.

If we consider referrals that were not added to the waiting list or provided treatment as “rejected”^3^, then 57.2% of children referred to CAMHS in Site A for suicidality were rejected, compared with 20.6% in Site B. These numbers highlight again the difference between outcomes for children referred to different services. The national CAMHS audit reports that 1 in 5 (20%) of referrals to CAMHS across Scotland are rejected (25). This study shows that while the number of rejected referrals for suicidal children in Site B is in keeping with this figure, in Site A they are more than double the reported national average. Neither service reject less referrals for reasons of suicide than the reported national average, which is contrary to a belief held by parents that CAMHS only see young people who are suicidal, as was reported in the audit of rejected referrals (15).

The study SIMD data demonstrates a difference in the proportion of referrals for children from areas of multiple deprivation between Site A and B, with more children in Site B being referred from areas of higher multiple deprivation. However, this may be reflective of the levels in deprivation across these sites more generally: more areas of Site B are among the 20% of most deprived areas in Scotland than in Site A (19% of data zones in Site B are considered in the lowest decile for deprivation, in Site A this figure is 8%). While there are pockets of areas of deprivation in Site A, poverty of access is a much more prominent issue, with almost half of localities reported as being in the lowest quintile for access (47%) (26, 27).

The 2018 audit of rejected referrals to CAMHS in Scotland (11) found there were a higher a number of rejected referrals for children from areas of multiple deprivation than more affluent areas. However, they were unable to situate this within the context of SIMD data for all CAMHS referrals, as this data is not routinely gathered or available. They suggest that higher numbers of referrals for children to CAMHS from deprived areas would be expected given what is known about the links between poverty and poorer mental health. There could be a disparity in access to mental health services for children based on social class (28). There are clearly established links between suicide and deprivation (29–31). Although postcode data may not provide the whole picture in relation to the adversity experienced by a child it is important their access to mental health support services are considered within a context of social stratification if we are to understand the specific barriers and challenges such as means of transport to attend appointments, that they face.

In both regions there were slightly higher numbers of referrals for females than males. However, the number of males referred averaged 41% across both regions (40.3% in Site A, and 42.2% in Site B), and there were much higher rates of self-harming behavior among boys generally [rates of self-harm have been found to be three times higher for girls than for boys (32)]. Given that completed suicide is known to be higher among young men and males generally, it could be a positive that boys are seeking help with suicidal feelings at a young age (1, 5, 33–36). However, this finding also highlights the importance of children receiving timely help and the opportunity that it is presented to provide an intervention at the point of referral. The age of children referred to both services ranged from 5–17yrs. Suicidality is generally perceived as an adult problem. While there is growing recognition that suicidality is an issue for many adolescents and young adults it is not commonly associated with younger children (37, 38). This study found that approximately 33% of all children who were referred due to suicidality were aged 12 or younger (30% in Site A; 35% in Site B).

It is debated whether children under the age of 12 fully comprehend suicide (39). Some evidence suggests they do present with suicidal ideation, make attempts to end their life, and complete suicide (38, 40). However, there remains a paucity of research regarding very young children and suicide (41). But given the increasing numbers of deaths by suicide among this population (33, 35), it may be harmful to ignore or dismiss young children presenting with suicidality because of a belief that they are too young to fully understand what suicide really means (42).

The underlying issues identified within the referrals in both health board areas have recognizable similarities. For example, 37.5% of children in Site A, and 41.1 % in Site B noted parental separation. Domestic abuse was reported in 8.3% of referrals in Site B, and 14% in Site A. Child abuse (physical, emotional, sexual or neglect) was mentioned in 12% of referrals in Site A, while the overall rate of child abuse within the referrals in Site B was 18 %, with child sexual abuse specifically mentioned in 10% of identified referrals. This could be because CAMHS in Site B have a dedicated service to support trauma recovery in children who have experienced sexual abuse, encouraging referrers to explicitly mention this.

Parental separation featured in approximately 40% (38% in Site A; 41% in Site B) of all referrals for children who were suicidal, highlighting this is a difficult issue for children not just at the point of separation but also after. It may be this is the case for all referrals to CAMHS and not just those identified for suicidality. An accurate number of children having experienced parental separation across Scotland is not available, however information from the 2011 census tells us that 31% of families with dependent children were lone parent households, 15% were cohabiting, and 54% were married. Of the cohabiting families 29% were stepfamilies, and 8% of married families were stepfamilies (43). This suggests that parental separation across the population of children in Scotland is perhaps not that different from the prevalence of parental separation in children referred to CAMHS for suicidality. Additionally, as is reported elsewhere (6, 37), suicidality in children generally stems from a combination of more than one issue and is not solely attributable to parental separation.

Approximately 20% of all children referred in this study for suicidality either had an autistic spectrum disorder (ASD) diagnosis, or ASD was queried within the referral. We know from the research literature generally that the links between ASD and suicide have been established (37, 44). Specialist support around suicidality should be made available for children with autism and their families.

Drugs and / or alcohol were only mentioned in 9% of referrals in Site A, and 11.1% in Site B. This supports the findings of other research in this area that suggest that unlike in adult populations there is not a clear association between suicidality and drugs / alcohol in children (6, 45, 46).

The data sets from Site A and B were different in that other mental health or neurological conditions (Low mood, anxiety, eating disorders, psychosis etc.) were only mentioned in 6.7% of referrals for children presenting with suicidality in Site B compared to 32% in Site A. This could be due to differences in the choice of language used by referrers to describe symptoms and feelings e.g., anxiety and low mood, and warrants further exploration in future studies. It could also be because the existence of the dedicated suicide and self-harm team in Site B means that rightly or wrongly referrers did not feel the need to pathologize mood and anxiety as much as they are more confident that the expression of suicidality alone meets the threshold for CAMHS. Additionally, referrals where the primary reason for referral was related to ASD were not screened in Site B as these were directed to another team. Importantly though these figures show that a sizeable proportion of the referrals in Site A suggest there is a co-occurring presenting mental health issue that may require assessment / treatment / support.

The underlying issues identified in these referrals supports what is already known about risk factors and suicide in children and young people (46). These are issued faced by many young people growing up. However, as was identified in the UK National Confidential Enquiry report, 2017 (6), young people who are suicidal often face multiple challenges, and it may be unhelpful to attempt to compartmentalize support around particular issues for individuals who are actively suicidal.

The problem, as it is presented in government reports (14, 15, 47), and was found in this study, suggests that CAMHS do not have the capacity to meet the needs of the numbers of children being referred. In Site A, most children who are referred for suicidality are not assessed or offered treatment. While in Site B, they have a dedicated team for children and young people who self-harm or are suicidal, and consequently assess 87% of all children referred for suicidality (48) argues that demand increases in line with service provision; therefore creation of specialist services to extend capacity results in increasing referrals as awareness of the service extends. However, this study did not find this to be the case. Referrals for children who presented as suicidal were approximately one quarter of all CAMHS referrals in both regions, although one had a specialist suicide and self-harm team.

### Strengths and limitations

This study is the first to present data on the numbers of children referred to CAHMS presenting as suicidal and the outcomes of their referrals. Identifying referrals for children who were suicidal from individual records was both a complex and arduous task, given the inconsistencies in recording practices and variation in referral information provided. There is an unavoidable element of interpretation, and construction as the qualitative referral information and record of first contact is deciphered and coded. This may have been approached differently by another researcher, what is presented here is a transparent report of the findings employing our pre-defined protocol.

Although, the sample size was small and any relationships between referral outcomes and demographic indicators difficult to ascertain, what is presented offers previously unknown insight into this important issue. This study was concerned with ascertaining the numbers of referrals for children to CAMHS for reasons of suicidality, and the broad outcomes of these referrals. The data extracted was limited to predefined variables relating to this purpose, however, it may have been helpful to also capture the nature and types of treatments offered. Additionally, although data collection was limited by the availability and accuracy of the data held by CAMHS, this highlights issues in recording practices and referral information requested.

## Conclusions

This study shows despite differences in geography and context, suicidality in children is a factor in approximately a quarter of all referrals to these two CAMHS, and there are vast differences in how these referrals are processed and responded to. There is little difference in the issues being identified by referrers, the age range of children, and the behaviors they present and yet there were very different outcomes, and pathways of care.

Given that 33% of referrals were for children under 12 years of age this highlights the often-missed opportunity for early intervention with very young suicidal children when they are not seen or offered support from CAMHS.

The data presented here is novel and will provide a vital source of information to decision makers and service providers in their consideration of service structures and allocation of resources. With growing numbers of referrals to CAMHS, and excessively long waiting times in many areas, it is vital that those identified as being at risk of suicide be provided with clear and consistent pathways of care.

Routine systems for collecting CAMHS data should include suicidality if we are to better understand the extent of this problem, and responses to referrals for suicidality from CAMHS. Further research is also needed to establish how this care journey is experienced by suicidal children and young people, and whether when they do receive treatment from CAMHS it meets their needs.

## Data availability statement

The datasets presented in this study can be found in online repositories. The names of the repository/repositories and accession number(s) can be found below: dataSTORRE https://datastorre.stir.ac.uk.

## Author contributions

LG conceived the idea for the study, extracted and input the data, and performed the analysis. LG, MM, and ED developed the study protocol and input to ethics and other approvals for access to the data. CB verified the analytical methods and results. MM and ED encouraged and supervised all stages of this work. All authors discussed the results and contributed to the final manuscript.

## Funding

LG conducted this study as part of an ESRC funded Ph.D.

## Conflict of interest

The authors declare that the research was conducted in the absence of any commercial or financial relationships that could be construed as a potential conflict of interest.

## Publisher's note

All claims expressed in this article are solely those of the authors and do not necessarily represent those of their affiliated organizations, or those of the publisher, the editors and the reviewers. Any product that may be evaluated in this article, or claim that may be made by its manufacturer, is not guaranteed or endorsed by the publisher.

## Footnotes

^1^Scotland has the highest rate of deaths by suicide across all age groups (2, 5).

^2^The actual number of children referred to CAMHS in Site B overall, would also include those referred for ASD assessments, the learning disabilities team, and psychological services and would be much higher. These referrals were not included or screened, on the advice of the data managers as these are not considered core CAMHS services, and the primary reason for their referral would not have been suicide.

^3^Rejected is the term used by the team conducting the national CAMHS audit (25), although redirected is often a preferred term.

## Nomenclature

For the purposes of clarity, the authors have used the following definitions:

- Suicidal behavior: Any form of self-harming behavior motivated by suicidal intent. It may have a fatal or non-fatal outcome.
- Suicidal ideation: Having thoughts about suicide, which may include planning suicide, sending, or writing suicide notes.
- Suicidality: A term of reference that includes both suicidal behavior and ideation.
- Self-harm: Any behavior that causes self-injury, with and without suicidal intent. Most people who self-injure do not intend die and differentiate between self-harming and suicidal behaviors.
