# Supplementary material for: Characteristics and outcomes of referrals to CAMHS for children who are thinking about or attempted suicide: A retrospective cohort study in two Scottish CAMHS
Source: Front Psychiatry. 2022 Sep 1;13:914479. doi: 10.3389/fpsyt.2022.914479 (PMC9477378; doi:10.3389/fpsyt.2022.914479)
Supplement: Supplementary file 1 [file Table_1.DOCX]

Retrospective Cohort Study - Table of variables extracted.

| Variable | Justification for extraction | Storage |
| --- | --- | --- |
| Name | These data were solely used to identify potential participants for anoth. | This data was stored in an excel spreadsheet on a password protected NHS computer within the NHS site. It was not removed from the site. |
| Address |  |  |
| Parent / carers name |  |  |
| Parent / carers address. |  |  |
| Reason for referral in relation to suicidality | This helped to identify any potential relationship between the presenting suicidal behaviour and referral outcome. | This data was input into an SPSS spreadsheet, on a password protected and encrypted University of Stirling Laptop. It was the transferred to the researchers University of Stirling password protected research drive. |
| Month of referral | This helped to establish the time from point of the referral to treatment. However, keeping to the month rather than date makes the individual less identifiable. |  |
| Occupation / Position of Referrer | This data was not linked to specific locations, or individuals minimising the potential for identification. This was used to establish if there is any potential relationship between the occupation of the referrer and the referral outcome. |  |
| Outcome of referral | This relates to whether the patient was seen / signposted on / on waiting list etc. This information was vital to address the research question. |  |
| Status of Referral | This information helped to describe the referral outcomes for the children – for example how many were on a waiting list. |  |
| Offered an Assessment | This information was important to show what happened after a referral was made. |  |
| SIMD 16 code | This was noted to be able to explore whether there is any relationship between postcode area and referral outcome – not as an identifier. |  |
| Ethnicity | This will be used to explore any differences in referral outcomes related to ethnicity. |  |
| Family circumstances | This was used to explore any differences in referral outcomes related to family circumstances. |  |
| Siblings | This was used to explore any differences in referral outcomes related to family circumstances. |  |
| Other issues identified by the referrer (e.g. Bullying, child sexual abuse, neglect, physical abuse, domestic violence, bereavement, identity, sexuality, ASD (Autism Spectrum Disorder), other mental health issues). | This was used to explore any differences in referral outcomes related to other underlying issues that were identified by the referrer. |  |
